# Expression of Cancer Stem Cell Markers EpCAM and CD90 Is Correlated with Anti- and Pro-Oncogenic EphA2 Signaling in Hepatocellular Carcinoma

**DOI:** 10.3390/ijms22168652

**Published:** 2021-08-11

**Authors:** Nobuhiko Asakura, Naotoshi Nakamura, Atsushi Muroi, Yosui Nojima, Taro Yamashita, Shuichi Kaneko, Kazuki Ikeda, Naohiko Koshikawa, Takashi Suzuki

**Affiliations:** 1 Center for Mathematical Modeling and Data Science, Osaka University, Osaka 580-8531, Japan; asakura@sigmath.es.osaka-u.ac.jp (N.A.); n-nakamura@sigmath.es.osaka-u.ac.jp (N.N.); nojima@sigmath.es.osaka-u.ac.jp (Y.N.); 2 Kanagawa Cancer Center Research Institute, Yokohama 241-8515, Japan; muroia@gancen.asahi.yokohama.jp; 3 Department of General Medicine, Kanazawa University Hospital, Kanazawa 920-8641, Japan; taroy@m-kanazawa.jp; 4 Department of Gastroenterology, Kanazawa University Hospital, Kanazawa 920-8641, Japan; skaneko@m-kanazawa.jp; 5 Department of Life Science and Technology, Tokyo Institute of Technology, Yokohama 226-8501, Japan; ikeda.k.bh@m.titech.ac.jp

**Keywords:** hepatocellular carcinoma, reverse phase protein array, liver cancer stem cells, EpCAM, CD90, EphA2, AKT

## Abstract

Hepatocellular carcinoma (HCC) is the third leading cause of cancer death worldwide. Additionally, the efficacy of targeted molecular therapies with multiple tyrosine kinase inhibitors is limited. In this study, we focused on the cellular signaling pathways common to diverse HCC cells and used quantitative reverse phase protein array (RPPA) and statistical analyses to elucidate the molecular mechanisms determining its malignancy. We examined the heterogeneity of 17 liver cancer cell lines by performing cluster analysis of their expression of CD90 and EpCAM cancer stem cell markers. Gaussian mixture model clustering identified three dominant clusters: CD90-positive and EpCAM-negative (CD90+), EpCAM-positive and CD90-negative (EpCAM+) and EpCAM-negative and CD90-negative (Neutral). A multivariate analysis by partial least squares revealed that the former two cell populations showed distinct patterns of protein expression and phosphorylation in the EGFR and EphA2 signaling pathways. The CD90+ cells exhibited higher abundance of AKT, EphA2 and its phosphorylated form at Ser^897^, whereas the EpCAM+ cells exhibited higher abundance of ERK, RSK and its phosphorylated form. This demonstrates that pro-oncogenic, ligand-independent EphA2 signaling plays a dominant role in CD90+ cells with higher motility and metastatic activity than EpCAM+ cells. We also showed that an AKT inhibitor reduced the proliferation and survival of CD90+ cells but did not affect those of EpCAM+ cells. Taken together, our results suggest that AKT activation may be a key pro-oncogenic regulator in HCC.

## 1. Introduction

Liver cancer is the sixth most commonly diagnosed cancer and the third leading cause of cancer-related deaths worldwide. Hepatocellular carcinoma (HCC) is the most prevalent type of primary liver cancer, accounting for 75–85% of liver cancer cases [1]. Even with the three pillars of HCC treatment (hepatectomy, local ablative therapies and transcatheter arterial chemoembolization), the rate of cancer recurrence is extremely high, as compared with other cancers, with recurrence rates of approximately 70% to 80% over 5 years. The prognosis for HCC patients is poor, with a low 5-year survival rate of less than 20% [2,3]. Sorafenib, a tyrosine kinase inhibitor (TKI), is the first-line treatment for advanced-stage HCC [4,5,6]. However, it only provides a modest median survival benefit of 2–3 months. Furthermore, its effectiveness is limited to approximately 30% of HCC patients. Most patients acquire sorafenib resistance within 6 months [7]. In recent years, additional TKIs, lenvatinib [8,9,10] and regorafenib [11,12] have been approved as first- and second-line treatments for advanced HCC, respectively. However, their therapeutic response and survival benefits are still low and limited.

This limited efficacy of TKIs for HCC most likely stems from tumor heterogeneity. This heterogeneity is proposed to arise from a subset of cancer stem cells (CSCs) with self-renewal and differentiation capabilities [13]. Liver CSCs are known to express specific cell surface markers, such as EpCAM, CD90 (Thy1), CD133, CD24 and CD44. Yamashita et al. [14] revealed that these CSCs are heterogeneous in their morphology, tumorigenicity and metastasis. Notably, EpCAM-positive HCC cells have an epithelial cell morphology and are highly tumorigenic but poorly metastatic. In contrast, CD90-positive HCC cells have a mesenchymal cell morphology and are highly metastatic but poorly tumorigenic. Furthermore, EpCAM-positive and CD90-positive HCC cells have differential sensitivity to various chemotherapeutic and targeted molecular agents, including sorafenib [14,15,16,17,18]. Thus, elucidating the regulatory mechanisms underlying these CSCs may help in a deeper understanding of HCC tumorigenicity, metastasis and drug resistance, as well as facilitate the development of more effective chemo- and targeted molecular therapies for advanced HCC.

Several signaling pathways have been implicated in the pathogenesis of HCC [19,20]. The epidermal growth factor receptor (EGFR) signaling cascade is one of the main signaling pathways involved in HCC tumorigenesis [21]. Activation of EGFR leads to downstream activation of the ERK/MAPK and PI3K/AKT/mTOR pathways. The ERK/MAPK pathway is involved in cell proliferation and differentiation. The PI3K/AKT/mTOR pathway is crucial for cell survival and growth and is involved in the regulation of cell motility and metastasis [22]. EGFR is overexpressed in a variety of malignant tumors, including HCC. Its inhibitors have been successfully used to treat breast, lung, colorectal and pancreatic cancers. However, for patients with advanced HCC, EGFR inhibitors show only modest clinical activity [20]. This raises the issue of HCC tumor heterogeneity likely to be due to CSCs; thus, the need to elucidate the regulatory mechanisms responsible for modulating the function of the EGFR signaling cascade should be considered.

Ephrin type A receptor 2 (EphA2) is a receptor tyrosine kinase that has been found to be overexpressed in a variety of cancers, including HCC [23]. Increased expression of EphA2 is associated with poor patient prognosis in HCC [24,25]. Intriguingly, EphA2 can cross-talk with EGFR and its downstream signaling pathways [26]. Furthermore, EphA2 signaling can be either anti- or pro-oncogenic, depending on ligand binding [27,28,29]. Binding of ephrin-A to EphA2 induces autophosphorylation of tyrosine residues and recruits p120-GTPase activating protein (Ras-GAP) to the plasma membrane, thereby suppressing the activation of the ERK/MAPK and PI3K/AKT/mTOR signaling pathways. Thus, ligand-dependent EphA2 signaling is anti-oncogenic. In contrast, when unliganded, EphA2 activation stimulates the ERK/MAPK and PI3K/AKT/mTOR signaling pathways. Activated AKT or RSK, a downstream kinase of the ERK/MAPK cascade, can phosphorylate EphA2 on the serine residue at 897 (Ser^897^). This, in turn, activates Rho GTPase signaling, resulting in cell migration and metastasis. Thus, ligand-independent activation of EphA2 has pro-oncogenic functions. The relative balance between these two types of EphA2 activation may be a key determinant of tumor heterogeneity. However, the mechanisms underlying ligand-dependent and ligand-independent EphA2 signaling have not been fully explored in HCC; hence, their connections to liver CSCs are largely unknown.

In this study, we classified 17 liver cancer cell lines based on the expression of EpCAM and CD90 CSC markers. Additionally, we compared the patterns of protein expression and phosphorylation of the EGFR and EphA2 signaling pathways between EpCAM-positive and CD90-positive cells. We found that pro-oncogenic EphA2 signaling was more pronounced in CD90-positive cells, in that they exhibited higher levels of EphA2, pEphA2-Ser^897^ and AKT. We further demonstrated that an AKT inhibitor selectively affected CD90-positive cell proliferation and survival.

## 2. Results

### 2.1. EpCAM and CD90 Expression Classify Hcc Cells into Functionally Distinct Clusters

**Figure 1:**
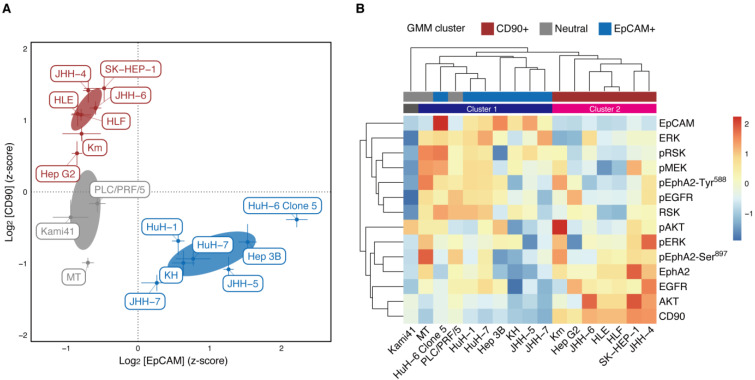
Clustering of 17 HCC cell lines with protein expression and phosphorylation. (**A**) GMM clustering of the cell lines based on the RPPA analysis of EpCAM and CD90. A VEV model (ellipsoidal, equal shape) with three components (clusters) was selected using BIC. The clusters are colored by red (CD90+), blue (EpCAM+) and grey (Neutral). Each point is the mean of six replicates. Error bars represent the SDs. Error ellipses denote the 1 σ regions of mixture Gaussian components. (**B**) Heat map and hierarchical clustering of the cell lines and all the RPPA analysis. Average linkage and Euclidean distance were used to create the dendrograms. The clustering result is depicted by colored bars (dark blue, Cluster 1; magenta, Cluster 2). The GMM clusters are also depicted by bars with the same color as (**A**).

To address the heterogeneity of HCC cells in terms of CSCs, we first applied Gaussian mixture model (GMM) clustering to examine the potential clusters in 17 liver cancer cell lines based on EpCAM and CD90 expression, as detected on the RPPA analysis (Figure S1). The clustering revealed three dominant HCC cell clusters (Figure 1A): EpCAM-negative and CD90-positive HCC cells (hereinafter ‘CD90+ cell cluster’), EpCAM-positive and CD90-negative HCC cells (‘EpCAM+ cell cluster’) and EpCAM-negative and CD90-negative HCC cells (‘Neutral cell cluster’). We did not find HCC cells with high expression of both EpCAM and CD90. The results confirmed the previously described pattern of mutually exclusive expression of EpCAM and CD90 in HCC cell lines [14]. Additionally, they are almost consistent with the following cell classifications [14]: HuH-1, HuH-7 and Hep 3 B were EpCAM+ cell lines; HLE, HLF and SK-Hep-1 were CD90+ cell lines; PLC/PRL/5 was a CD90+ cell line, but contained only a small population of CD90+ cells.

Next, we conducted RPPA analysis of protein expression and phosphorylation in the EGFR and EphA2 signaling pathways (EGFR, pEGFR, MEK, pMEK, ERK, pERK, RSK, pRSK, AKT, pAKT, EphA2, pEphA2-Tyr^588^ and pEphA2-Ser^897^. Here, ‘p’ denotes phosphorylated proteins) for 17 liver cancer cell lines. Then, using this data and RPPA data for EpCAM and CD90, we performed hierarchical clustering of those cell lines (Figure 1B, column-wise). In this clustering, the cell lines were classified into Clusters 1 and 2, with the exception of Kami41. Cluster 1 corresponded to the EpCAM+ and neutral cell clusters, whereas Cluster 2 coincided with the CD90+ cell cluster. The consistent clustering result proved the validity of the GMM clustering with EpCAM and CD90; therefore, it was used in the analysis below.

### 2.2. Protein Expression and Phosphorylation Specific to Cd90+ and Epcam Cell Clusters

**Figure 2:**
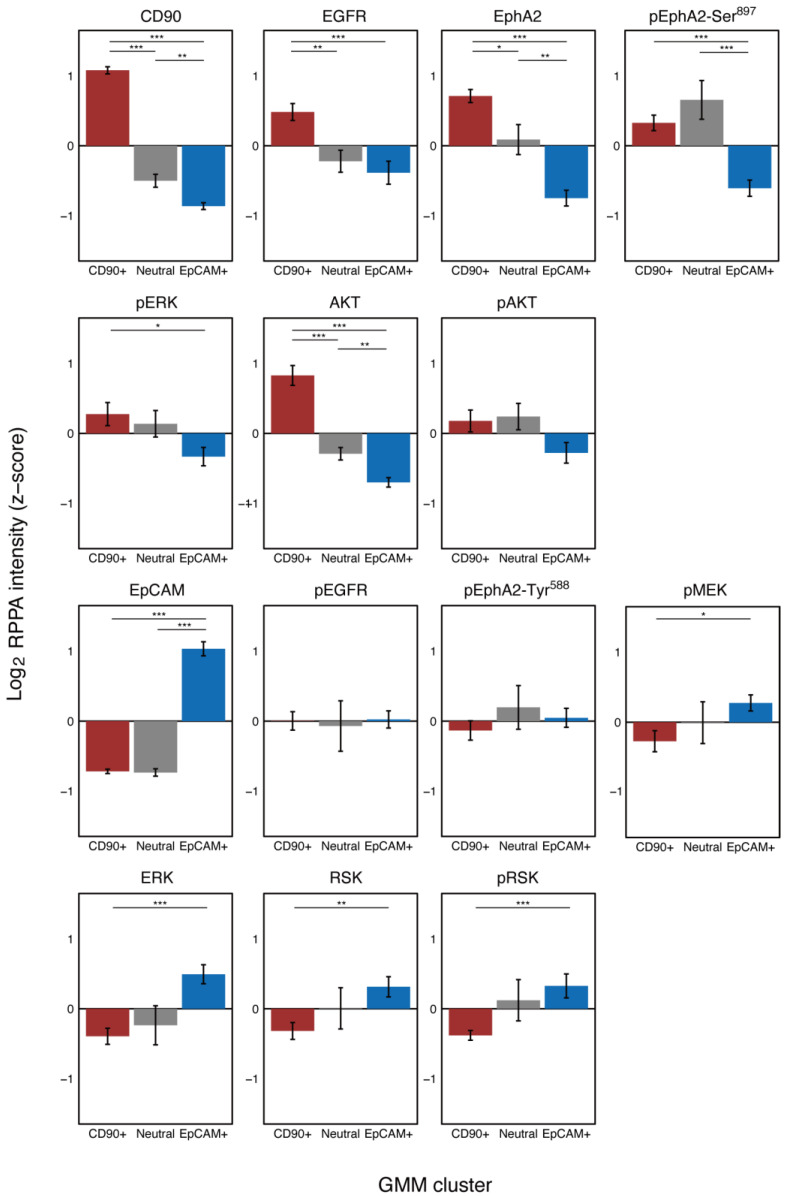
Comparison of protein expression and phosphorylation among the different GMM clusters. Bars represent the means ± SD of the GMM cluster cells. For all the proteins and phosphoproteins except for pAKT, pEGFR and pEphA2-Tyr^588^, the GMM cluster had a significant effect on the RPPA analysis (*p* < 0.05, Welch’s ANOVA with multiple correction). For the significant proteins and phosphoproteins, a Games-Howell post-hoc test was used for pairwise comparisons. * *p* < 0.05, ** *p* < 0.01, *** *p* < 0.001.

Results of the hierarchical clustering of protein expression and phosphorylation (Figure 1B, row-wise) reveal that EpCAM is in the same cluster as ERK, RSK and CD90, as AKT. In fact, ERK and RSK seem to be overexpressed in the EpCAM+ cell cluster and AKT in the CD90+ cell cluster. To statistically show this, we used one-way analysis of variance (ANOVA) followed by multiple comparisons to analyze protein expression and phosphorylation specific to each cell cluster (Figure 2). We found that EGFR, AKT and EphA2 were significantly overexpressed in the CD90+ cell cluster; pEphA2-Ser^897^ was significantly overexpressed in both the CD90+ and Neutral cell clusters. High expression of these proteins leads to malignancy via ligand-independent, oncogenic EphA2 signaling and is consistent with known phenotypes of the CD90+ cell cluster [14]. In contrast, pMEK, ERK, RSK and pRSK in the EGFR signaling pathway were significantly overexpressed in the EpCAM+ cell cluster. ERK is known to indirectly inactivate GSK-3β [30] and enhance sorafenib sensitivity in HCC [31]. Thus, the result is in agreement with the high sorafenib sensitivity of the EpCAM+ cell cluster [32].

### 2.3. Partial Least Squares Analysis Reveals Anti- and Pro-Oncogenic Activity of Epcam+ and Cd90+ Cells

We further examined the differential expression of proteins and phosphoproteins among the GMM clusters using partial least squares (PLS) analysis [33,34,35]. PLS is a multivariate technique similar to principal component analysis (PCA), with the exception that PLS extracts latent variables (LVs) that maximize the covariance between independent and dependent datasets using singular value decomposition. Thus, when applied to the current data, it seeks to find LVs that maximize the covariance between the RPPA data and the GMM clusters. These LVs are likened to the factor scores in the PCA. The LVs also have a counterpart of factor loadings in PCA, a pair of singular vectors, or *saliences*. The relative contribution of each LV to the covariance is assessed through a singular value, which is the square root of an eigenvalue in the PCA. As there are three GMM clusters, the number of LVs to be obtained is at most two (equivalent to the number of independent orthogonal contrasts for the three groups).

**Figure 3:**
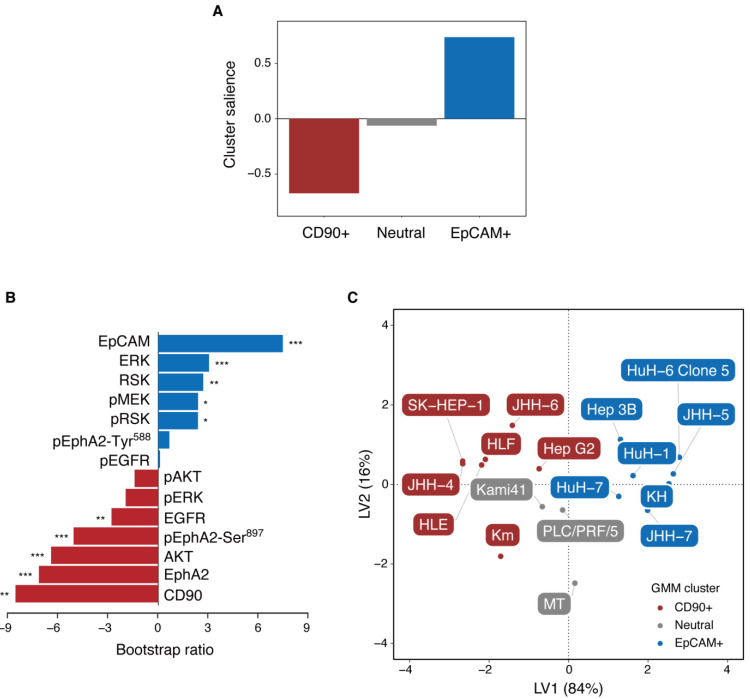
Mean-centered PLS analysis of the RPPA data. (**A**) Bar graph of cluster salience for the significant latent variable (LV1). The significance was assessed using 1000-fold permutation tests. (**B**) Bootstrap ratio (BSR) of the protein salience for LV1. The BSR is the ratio of salience to its bootstrap estimated SE, which approximates a z-score. The depicted BSRs are based on 1000 bootstrap resampling. * |BSR| > 1.96 (*p* < 0.05), ** |BSR| > 2.58 (*p* < 0.01), *** |BSR| > 3.03 (*p* < 0.001). (**C**) Score plot of LVs for the RPPA data.

Using a mean-centered PLS [33], we found only one significant latent variable (LV1) that accounted for 84% of the covariance (1000-fold permutation tests, *p* < 0.0001). Figure 3A shows the cluster salience, which corresponds to the optimal contrast between GMM clusters that accounts for variation in the RPPA data. This indicates that the current data are best characterized by contrasting activation between CD90+ and EpCAM+ cells. In addition, the fact that the salience of the Neutral cell cluster is almost zero indicates that Neutral cells do not have a common pattern of protein expression and phosphorylation into which they are clustered. This means that they are heterogeneous in their protein expression and phosphorylation, which explains why hierarchical clustering did not reveal a distinct cluster for Neutral cells (Figure 1B).

Figure 3B depicts the protein salience, which illustrates the pattern of protein expression and phosphorylation that optimally differentiates the GMM clusters as identified in the cluster salience (i.e., between the CD90+ and EpCAM+ clusters). Negative salience indicates the proteins and phosphoproteins that are more abundant in CD90+ cells than in EpCAM+ cells; positive protein salience indicates those that are more abundant in EpCAM+ cells than in CD90+ cells. The results are in agreement with those obtained from the univariate analysis (Figure 2), excepting that pERK level was not statistically significant. This indicates that when adjusted by multivariate analysis, the contribution of pERK was not large enough to distinguish between the CD90+ and EpCAM+ clusters. Thus, we found that CD90+ cells exhibited significantly higher levels of AKT, EphA2 and pEphA2-Ser^897^, which is consistent with the upregulation of pro-oncogenic, ligand-independent EphA2 signaling. In contrast, EpCAM+ cells exhibited significantly higher levels of ERK, RSK and pRSK, which are involved in MAPK signaling. They also exhibit modest, but not significant, activation of pEphA2-Tyr^588^, which is reminiscent of anti-oncogenic, ligand-dependent EphA2 signaling. These results suggest that anti-oncogenic EphA2 signaling may occur in EpCAM+ cells, but appears too weak to suppress pro-oncogenic MAPK signaling.

Figure 3C shows the PCA style score plot of LVs for the RPPA data. In fact, LV1 is a vector representing how strongly each cell exhibits a pattern of protein expression and phosphorylation, as indicated by the protein salience depicted in Figure 3B. LV2 was similarly derived from the other protein salience (not shown). The figure shows that LV1 separates CD90+, EpCAM+ and Neutral cells from each other. However, they were less clustered and more broadly distributed than those plotted by EpCAM and CD90 expression (Figure 1A). This implies that the heterogeneity of HCC is likely to be described as a spectrum in which the relative balance between anti- and pro-oncogenic EphA2 signaling can vary.

### 2.4. AKT Inhibitor Suppressed Cd90+ Cell Proliferation

**Figure 4:**
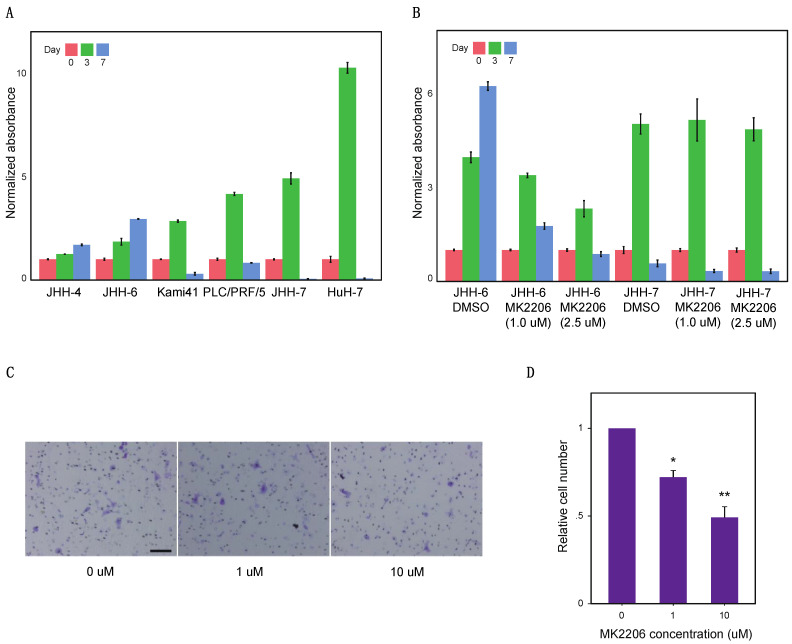
Cell proliferation and migration assays. (**A**) Proliferation of six representative HCC cell lines. For each cell line, absorbance data were normalized with respect to the mean of Day 0 (*n* = 3). The significant effect of Day was confirmed for all the cell lines (*p* < 0.0001, Welch’s ANOVA) and every pairwise comparison between Days was significant in each cell line (*p* < 0.05, Games-Howell post-hoc test). (**B**) The effect of the AKT inhibitor MK2206 on the proliferation of JHH-6 and JHH-7. For each cell line, absorbance data were normalized with respect to the mean of Day 0 (*n* = 3). Significant effects of Day, AKT inhibitor and their interaction (*p* < 0.0001, two-way ANOVA) were confirmed in JHH-6; only a significant effect of Day (*p* < 0.0001, two-way ANOVA) was confirmed in JHH-7. (**C**) JHH-6 cells were seeded in a migration chamber with the medium containing 0.5% FBS and indicated concentrations of MK2206. After 4 h of incubation, the migrated cells were stained with either 0.25% crystal violet/20% methanol and counted on microscopy (10× magnification). Scale bar: 100 μm. (**D**) Transwell migration of JHH-6 cells was suppressed by MK2206 in a dose-dependent manner. The data were normalized for each experiment with respect to that of control cells (0 μM). The plot shows the mean + SD of three independent experiments. The significance was assessed using one-sample t-test with Bonferroni correction. * *p* < 0.05, ** *p* < 0.001.

Having identified AKT as a potential molecular target specific to the CD90+ cell cluster, we evaluated the effect of the AKT inhibitor, MK2206, on cancer cell proliferation. Using two cell lines from each cell cluster (JHH-4 and JHH-6 from the CD90+ cell cluster; Kami41 and PLC/PRF/5 from the Neutral cell cluster; JHH-7 and HuH-7 from the EpCAM+ cell cluster), we first compared cell proliferation among these clusters under low-serum culture conditions (Figure 4A). When we started culturing at 5,000 cells/well on day 0, all cell lines significantly proliferated until day 3. In particular, the Neutral and EpCAM+ cell clusters showed higher cell proliferation up to day 3, as compared with the CD90+ cell cluster. However, the former clusters could not survive until day 7, whereas the latter cluster continued proliferating from day 3 to day 7. This indicates the capacity of the CD90+ cell cluster to proliferate in an anchorage-independent manner under high cell density conditions.

Noting this marked difference in cell proliferation between the CD90+ and EpCAM+ cell clusters, we next examined the effect of AKT inhibition on cell proliferation in the CD90+ cell cluster by comparing it with that in the EpCAM+ cell cluster. Both JHH-6 (CD90+ cell cluster) and JHH-7 (EpCAM+ cell cluster) were treated with MK2206 under the same culture conditions (Figure 4B). For JHH-6, two-way ANOVA revealed significant main effects of day, AKT inhibitor and their interaction (*p* < 0.0001). This indicates that the inhibitor changed the pattern of proliferation time course from a monotonic increase to an inverted V-shape, making it similar to that of EpCAM+ cells without the inhibitor. Thus, MK2206 suppressed the growth of JHH-6 from day 3 to day 7. In contrast, for JHH-7, two-way ANOVA revealed only a significant main effect of day (*p* < 0.0001). This suggests that the inhibitor did not affect the proliferative ability of JHH-7 cells. Overall, these results suggest that the effect of AKT inhibition on proliferation is specific to the CD90+ cell cluster.

### 2.5. AKT Inhibitors Suppress Cd90+ Cell Migration

We also examined the effect of AKT inhibitor on cell motility using a transmigration chamber (Figure 4C,D). JHH-6 cells seeded at 20,000 cells/well in the upper chamber migrated to the lower chamber in growth medium containing 0.5% and 10% FBS, respectively. The number of migrating cells significantly decreased with the addition of MK2206 in a dose-dependent manner, demonstrating that AKT inhibition suppressed cell motility in the CD90+ cell cluster.

## 3. Discussion

The prognosis of HCC remains poor because of its high recurrence rate and drug resistance. Despite recent progress in targeted molecular therapies for advanced HCC, their clinical benefits are still limited. This probably stems from the tumor heterogeneity of HCC, which is linked to liver CSCs. In our study, 17 liver cancer cell lines were classified into clusters (EpCAM+, CD90+ and Neutral) according to their expression of CSC markers, EpCAM and CD90. This classification was congruent with their known phenotypes: EpCAM+ cells show typical epithelial morphology and high tumorigenicity, whereas CD90+ cells show mesenchymal cell morphology and high metastatic activity to distant organs in vivo [14].

Furthermore, we found that these CSC-positive cell clusters showed differential protein expression and phosphorylation in the EGFR and EphA2 signaling pathways. In particular, CD90+ cells exhibited higher levels of AKT, EphA2 and pEphA2-Ser^897^. High levels of AKT expression and pEphA2-Ser^897^ were consistent with our observation that these cells were able to survive under high cell density conditions. Additionally, pEphA2-Ser^897^ activates the small GTPase RhoG and enhances cell motility, invasion and metastasis [28] and is also involved in mediating anchorage-independent cell survival and resistance to anoikis [36]. Thus, CD90+ cells show enhanced pro-oncogenic, ligand-independent EphA2 signaling. In contrast, EpCAM+ cells exhibited higher levels of ERK, RSK and pRSK. Consistent with the activation of the ERK/MAPK pathway, these cells showed higher growth activity than CD90+ cells. In addition, the absence of activation of the PI3K/AKT/mTOR pathway explains the observation that they did not survive under high cell density conditions.

Finally, we demonstrated that the AKT inhibitor, MK2206, blocks the prolonged survival of CD90+ cells and reduces their motility. Previous reports have suggested that the drug inhibits the proliferation of some HCC cell lines [37,38,39,40]. Our results confirmed this theory and further established that inhibition works particularly on CD90+ cells. Importantly, CD90+ cells do not express conventional tumor markers for HCC, such as PIVKA-II or AFP. Recently, we reported that CD90+ HCC cells express monomeric laminin-γ2 (LG2m) as a tumor marker. Serum LG2m enables the detection of HCC, including CD90+ HCCs [41]. The development of targeted molecular therapies against CD90+ cells is an urgent task for the complete cure of HCC. When combined with existing TKIs, we speculate that AKT inhibitors are therapeutic agents that may potentially target HCC heterogeneity.

## 4. Materials and Methods

### 4.1. Antibodies

Monoclonal antibodies against EGFR (D38B1), phospho-EGFR (Tyr^1068^) (D7A5), phospho-MEK1/2-Ser^217/221^ (41G9), ERK1/2 (4696), phospho-ERK1/2-Thr^202^/Tyr^204^ (13.14.4E), RSK1/2/3 (32D), phospho-RSK1/2/3-Ser^380^ (D3H11), AKT (40D4), phospho-AKT-Ser^437^ (D9E), phospho-EphA2 -Tyr^588^ (D7 × 2L) and phospho-EphA2 -Ser^897^ (D9A1) were purchased from Cell Signaling Technology (Danvers, MA, USA). A monoclonal antibody against EphA2 (C-3) was purchased from Santa Cruz Biotechnology (Dallas, TX, USA). A polyclonal antibody against EpCAM (21050-1-AP) and a monoclonal antibody against CD90 (66766-1-Ig) were purchased from Proteintech (Rosemont, IL, USA). All antibodies were validated for RPPA analysis by western blot analysis (Figure S2).

### 4.2. Cell Lines and Culture Conditions

Nine human hepatocellular carcinoma (HCC) cell lines (JHH-4, JHH-5, JHH-6, JHH-7, HuH-1, HuH-7, PLC/PRF/5, HLE and HLF) and two hepatoblastoma cell lines (HuH-6 clone5 and Hep G2) were obtained from the JCRB Cell Bank (National Institute of Biomedical Innovation Health and Nutrition, Osaka, Japan). Additionally, KH, Km, Kami41 and MT cell lines were established at Kanazawa University [42,43,44]. Hep 3B and SK-Hep-1 cell lines were purchased from the American Type Culture Collection (ATCC, Manassas, VA, USA). JHH-4, JHH-7, HuH-1, HuH-7, PLC/PRF/5, HLE, HLF, SK-Hep-1, HuH-6 clone5, Hep G2 and Hep 3B cells were cultured in DMEM (Thermo Fisher Scientific, Waltham, MA, USA). JHH-5 and JHH-6 cells were cultured in William’s E medium (Thermo Fisher Scientific, Waltham, MA, USA). KH, Km, Kami41 and MT were cultured in collagen-coated plates in DMEM medium. All cells were cultured in a medium supplemented with 10% FBS at 37 °C with 5% CO_2_.

### 4.3. Reverse Phase Protein Array (RPPA)

Confluent liver cancer cells were cultured under serum-starved conditions for 24 h and were lysed using T-PER protein extraction reagent (Thermo Fisher Scientific), supplemented with inhibitors (100 mM NaF, 1mM Na3VO4, 10 mM NaPi-IIb, 1 mM EDTA, PhosSTOP (Sigma-Aldrich, Missouri, MO, USA) and protease inhibitor cocktails (1.04 mM AEBSF, 800 nM aprotinin, 40 µM bestatin, 14 µM E-64, 20 µM leupeptin, 15 µM pepstatin A, Sigma-Aldrich). The lysates were scraped from the culture dish, collected and centrifuged at 15,000× *g* at 4 °C for 20 min; the supernatant was subjected to the following analyses. After adjusting the protein concentration to approximately 1.0 mg/mL according to Bradford protein assay (Bio-Rad), the lysates were manually diluted in two-fold serial dilutions with an extraction buffer. The diluted lysates were boiled with 2% SDS and 2.5% β-mercaptoethanol and printed onto nitrocellulose-coated slides in four replicates (Grace Bio-Labs, Bend, OR, USA) using an Aushon Biosystems 2470 arrayer (Burlington, MA, USA). After blocking with an ODYSSEY blocking buffer (LI-COR Biosciences, Lincoln, NE, USA) supplemented with 0.1% Tween-20, the blotted slides were probed with validated primary antibodies, followed by secondary antibodies conjugated to infrared dyes, IRDye 680RD and 800CW (LI-COR Biosciences) (Figure S3). A total of six replicate slides were scanned using an ODYSSEY scanner (LI-COR Biosciences). The signal intensity of each spot was quantified using Image Studio (LI-COR Biosciences) according to the manufacturer’s instructions. For each slide, the RPPA data of protein expression and phosphorylation for each cell line were normalized by the RPPA intensity of house-keeping protein γ-tubulin, log2-transformed and z-score standardized across all cell lines.

### 4.4. Cell Growth and Migration Assays

JHH-6, JHH-7, Kami41, PLC/PRF/5, JHH-7 and HuH-7 cell lines were suspended in serum-free medium containing 0.5% fetal bovine serum, seeded into a 24-well culture plate (5 × 10^3^ cells/well; TPP Techno Plastic Products AG, Trasadingen, Switzerland) and incubated for 7 days at 37 °C in 5% CO_2_. Additionally, the JHH6 and JHH7 cell lines were further treated with either DMSO or MK2206 (Sigma) at the indicated concentrations and incubated for 7 days at 37 °C in 5% CO_2_. The number of living cells was counted using either a hemocytometer or Coulter Counter (Beckman Coulter, Inc., Pasadena, CA, USA). Transwell migration assays were performed as described previously [45]. Briefly, transwell inserts with 8 μm-sized filters (Falcon) were inserted into 24-well plates. Growth medium containing 10% FBS were added to the lower chamber, while a cell suspension (2 × 10^4^ cells) in growth medium containing 0.5% FBS was introduced into the upper chamber. The plates were incubated at 37 °C in 5% CO_2_ for 4 h. After incubation, the cells that had migrated to the lower side were stained with 0.25% crystal violet/20% methanol solution and counted using a light microscope at ×10 magnification. The values obtained represent the averages of the five fields.

### 4.5. Clustering Analysis

We performed Gaussian mixture model (GMM) clustering of 17 liver cancer cell lines based on the RPPA intensities for EpCAM and CD90. We used the R (version 4.0.3) package mclust [46] to fit the GMMs. The optimal number and shape (covariance matrix) of mixture Gaussian components (i.e., clusters) were selected using the Bayesian information criterion (BIC). We also performed hierarchical agglomerative clustering (average linkage, Euclidean distance metric) of the cell lines using all the measured protein expression and phosphorylation values.

### 4.6. Partial Least Squares Analysis

Partial least squares (PLS) analysis [33,34,35] is a multivariate statistical technique for extracting latent variables (LVs) that maximize the covariance between two datasets using singular value decomposition (SVD). We conducted a mean-centered PLS [33] to find LVs that optimally account for the covariance between the RPPA data and GMM clusters. The RPPA data were transformed into a 17 × 14 data matrix, in which the rows represent each of the GMM clusters for each cell line, while the columns represent the RPPA intensities for all the measured protein expression and phosphorylation. The data matrix was then within-cluster-averaged and column-wise mean-centered. The transformed Matrix M was then subjected to SVD: M = U∑V’. The columns of U and V (left and right singular vectors) are referred to as *saliences* in the PLS literature. The left singular vectors are basis vectors for the row space of M, representing independent, orthonormal contrasts among the GMM clusters (cluster saliences). The right singular vectors are basis vectors for the column space of M, representing independent, orthonormal patterns of protein expression and phosphorylation (protein salience). LVs are computed by projecting the original data matrix onto these saliences. The diagonal matrix ∑ provides the singular values, each representing the covariance between a pair of cluster and protein saliences.

The statistical significance of the LVs was assessed using permutation tests. Each permutation involves random reassignment of the order of the GMM clusters to each cell line by sampling without replacement. Each LV was considered significant when the actual singular value was larger than the permutated singular value in more than 95% of all permutations (corresponding to a threshold *p* < 0.05). In addition, the reliability of protein salience was assessed using the bootstrap estimation of standard errors (SEs). This entails random sampling with replacement of each cell line data, keeping the cluster membership fixed for each cell line. Bootstrapped SEs were used to calculate bootstrap ratios (BSRs; original saliences divided by bootstrapped SEs). The BSR is approximately equivalent to a z-score and can be used to define a threshold to assess significance (e.g., |BSR| > 1.96 corresponds to *p* < 0.05). We computed 1000 permutation tests and 1000 bootstrap estimations. We note that no corrections for multiple comparisons are necessary, as PLS analyses are conducted in a single mathematical step on the whole set of protein expression and phosphorylation.

### 4.7. Statistical Analysis

The differences in protein expression and phosphorylation among the GMM clusters were evaluated by Welch’s analysis of variance (ANOVA) with Benjamini–Hochberg multiple testing correction, followed by a post-hoc Games–Howell test. Moreover, the effect of incubation day on cell growth was evaluated for each cell line using Welch’s ANOVA with Games–Howell post-hoc test. The effects of incubation day and AKT inhibitor on cell growth were evaluated for each cell line by two-way ANOVA using a heteroscedasticity-corrected coefficient covariance matrix. The effect of the AKT inhibitor on cell migration was evaluated by a one-sample t-test (two-tailed) with Bonferroni correction.

## 5. Conclusions

In summary, liver cancer cell lines were distinctively clustered according to their expression of CSC markers, EpCAM and CD90. While EpCAM+ cells exhibited higher levels of ERK, RSK and pRSK, CD90+ cells exhibited higher levels of AKT, EphA2 and pEphA2-Ser^897^. This indicates that CD90+ cells show enhanced pro-oncogenic, ligand-independent EphA2 signaling. We further demonstrated that the AKT inhibitor MK2206 reduced the proliferation and survival of CD90+ cells. Our findings suggest that AKT activation may be a key pro-oncogenic regulator in HCC.

## Data Availability

Data is contained within the article or supplementary material.

## Supplementary Materials

The following are available online at https://www.mdpi.com/article/10.3390/ijms22168652/s1, Figure S1: RPPA analysis of protein expression and phosphorylation across 17 HCC cell lines, Figure S2: Antibodies used for RPPA were validated by Western blots, Figure S3: Captured images of RPPA analysis. Primary antibodies probed to target proteins in samples were detected using secondary antibodies conjugated to infrared dyes against rabbit (red) or mouse (green).

## References

[1] Sung H., Ferlay J., Siegel R.L., Laversanne M., Soerjomataram I., Jemal A., Bray F. (2021). Global cancer statistics 2020: GLOBOCAN estimates of incidence and mortality worldwide for 36 cancers in 185 countries. CA Cancer J. Clin..

[2] Bahardoust M., Sarveazad A., Agah S., Babahajian A., Amini N. (2019). Predictors of 5 year survival rate in hepatocellular carcinoma patients. J. Res. Med. Sci..

[3] Villanueva A. (2019). Hepatocellular Carcinoma. N. Engl. J. Med..

[4] Wilhelm S.M., Carter C., Tang L., Wilkie D., McNabola A., Rong H., Chen C., Zhang X., Vincent P., McHugh M. (2004). BAY 43-9006 Exhibits Broad Spectrum Oral Antitumor Activity and Targets the RAF/MEK/ERK Pathway and Receptor Ty-rosine Kinases Involved in Tumor Progression and Angiogenesis. Cancer Res..

[5] Llovet J.M., Ricci S., Mazzaferro V., Hilgard P., Gane E., Blanc J.F., De Oliveira A.C., Santoro A., Raoul J.L., Forner A. (2008). Sorafenib in Advanced Hepatocellular Carcinoma. N. Engl. J. Med..

[6] Cheng A.-L., Kang Y.-K., Chen Z., Tsao C.-J., Qin S., Kim J.S., Luo R., Feng J., Ye S., Yang T.-S. (2009). Efficacy and safety of sorafenib in patients in the Asia-Pacific region with advanced hepatocellular carcinoma: A phase III randomised, double-blind, placebo-controlled trial. Lancet Oncol..

[7] Zhu Y.-J., Zheng B., Wang H.-Y., Chen L. (2017). New knowledge of the mechanisms of sorafenib resistance in liver cancer. Acta Pharmacol. Sin..

[8] Padda I.S., Parmar M. (2021). Lenvatinib.

[9] Kudo M., Finn R.S., Qin S., Han K.-H., Ikeda K., Piscaglia F., Baron A., Park J.-W., Han G., Jassem J. (2018). Lenvatinib versus sorafenib in first-line treatment of patients with unresectable hepatocellular carcinoma: A randomised phase 3 non-inferiority trial. Lancet.

[10] Suyama K., Iwase H. (2018). Lenvatinib. Cancer Control.

[11] Wilhelm S.M., Dumas J., Adnane L., Lynch M., Carter C.A., Schütz G., Thierauch K.-H., Zopf D. (2011). Regorafenib (BAY 73-4506): A new oral multikinase inhibitor of angiogenic, stromal and oncogenic receptor tyrosine kinases with potent preclinical antitumor activity. Int. J. Cancer.

[12] Bruix J., Qin S., Merle P., Granito A., Huang Y.-H., Bodoky G., Pracht M., Yokosuka O., Rosmorduc O., Breder V. (2017). Regorafenib for patients with hepatocellular carcinoma who progressed on sorafenib treatment (RESORCE): A randomised, double-blind, placebo-controlled, phase 3 trial. Lancet.

[13] Yamashita T., Wang X.W. (2013). Cancer stem cells in the development of liver cancer. J. Clin. Investig..

[14] Yamashita T., Honda M., Nakamoto Y., Baba M., Nio K., Hara Y., Zeng S.S., Hayashi T., Kondo M., Takatori H. (2013). Discrete nature of EpCAM+and CD90+cancer stem cells in human hepatocellular carcinoma. Hepatology.

[15] Gao Q., Zhu H., Dong L., Shi W., Chen R., Song Z., Huang C., Li J., Dong X., Zhou Y. (2019). Integrated Proteogenomic Characterization of HBV-Related Hepatocellular Carcinoma. Cell.

[16] Li L., Zhao G.-D., Shi Z., Qi L.-L., Zhou L.-Y., Fu Z.-X. (2016). The Ras/Raf/MEK/ERK signaling pathway and its role in the occurrence and development of HCC. Oncol. Lett..

[17] Liu Y.-C., Yeh C.-T., Lin K.-H. (2020). Cancer Stem Cell Functions in Hepatocellular Carcinoma and Comprehensive Therapeutic Strategies. Cells.

[18] Yoshida M., Yamashita T., Okada H., Oishi N., Nio K., Hayashi T., Nomura Y., Hayashi T., Asahina Y., Ohwada M. (2017). Sorafenib suppresses extrahepatic metastasis de novo in hepatocellular carcinoma through inhibition of mesenchymal cancer stem cells characterized by the expression of CD90. Sci. Rep..

[19] Dimri M., Satyanarayana A. (2020). Molecular Signaling Pathways and Therapeutic Targets in Hepatocellular Carcinoma. Cancers.

[20] Whittaker S., Marais R., Zhu A.X. (2010). The role of signaling pathways in the development and treatment of hepatocellular carcinoma. Oncogene.

[21] Komposch K., Sibilia M. (2015). EGFR Signaling in Liver Diseases. Int. J. Mol. Sci..

[22] Zhou Q., Huang T., Wang Y.-F., Zhou X.-B., Liang L.-J., Peng B.-G. (2011). Role of tissue factor in hepatocellular carcinoma genesis, invasion and metastasis. Chin. Med. J..

[23] Fan M., Liu Y., Xia F., Wang Z., Huang Y., Li J., Wang Z., Li X. (2014). Increased expression of EphA2 and E-N cadherin switch in primary hepatocellular carcinoma. Tumori J..

[24] Cui X.-D., Lee M.-J., Yu G.-R., Kim I.-H., Yu H.-C., Song E.-Y., Kim D.-G. (2009). EFNA1 ligand and its receptor EphA2: Potential biomarkers for hepatocellular carcinoma. Int. J. Cancer.

[25] Iida H., Honda M., Kawai H.F., Yamashita T., Shirota Y., Wang B.-C., Miao H., Kaneko S. (2005). Ephrin-A1 expression contributes to the malignant characteristics of -fetoprotein producing hepatocellular carcinoma. Gut.

[26] Cioce M., Fazio V. (2021). EphA2 and EGFR: Friends in Life, Partners in Crime. Can EphA2 Be a Predictive Biomarker of Response to Anti-EGFR Agents?. Cancers.

[27] Koshikawa N., Hoshino D., Taniguchi H., Minegishi T., Tomari T., Nam S.-O., Aoki M., Sueta T., Nakagawa T., Miyamoto S. (2015). Proteolysis of EphA2 Converts It from a Tumor Suppressor to an Oncoprotein. Cancer Res..

[28] Miao H., Li D.-Q., Mukherjee A., Guo H., Petty A., Cutter J., Basilion J.P., Sedor J., Wu J., Danielpour D. (2009). EphA2 Mediates Ligand-Dependent Inhibition and Ligand-Independent Promotion of Cell Migration and Invasion via a Reciprocal Regulatory Loop with Akt. Cancer Cell.

[29] Zhou Y., Sakurai H. (2017). Emerging and Diverse Functions of the EphA2 Noncanonical Pathway in Cancer Progression. Biol. Pharm. Bull..

[30] Ding Q., Xia W., Liu J.-C., Yang J.-Y., Lee D.-F., Xia J., Bartholomeusz G., Li Y., Pan Y., Li Z. (2005). Erk Associates with and Primes GSK-3β for Its Inactivation Resulting in Upregulation of β-Catenin. Mol. Cell.

[31] Zhang S., Gao W., Tang J., Zhang H., Zhou Y., Liu J., Chen K., Liu F., Li W., To S.K.Y. (2020). The Roles of GSK-3β in Regulation of Retinoid Signaling and Sorafenib Treatment Response in Hepatocellular Carcinoma. Theranostics.

[32] Garten A., Grohmann T., Kluckova K., Lavery G., Kiess W., Penke M. (2019). Sorafenib-Induced Apoptosis in Hepatocellular Carcinoma Is Reversed by SIRT1. Int. J. Mol. Sci..

[33] Krishnan A., Williams L., McIntosh A.R., Abdi H. (2011). Partial Least Squares (PLS) methods for neuroimaging: A tutorial and review. NeuroImage.

[34] McIntosh A.R., Lobaugh N.J. (2004). Partial least squares analysis of neuroimaging data: Applications and advances. NeuroImage.

[35] McIntosh A.R., Bookstein F.L., Haxby J.V., Grady C.L. (1996). Spatial Pattern Analysis of Functional Brain Images Using Partial Least Squares. NeuroImage.

[36] Harada K., Hiramoto-Yamaki N., Negishi M., Katoh H. (2011). Ephexin4 and EphA2 mediate resistance to anoikis through RhoG and phosphatidylinositol 3-kinase. Exp. Cell Res..

[37] Simioni C., Martelli A.M., Cani A., Cetin-Atalay R., McCubrey J., Capitani S., Neri L.M. (2013). The AKT Inhibitor MK-2206 is Cytotoxic in Hepatocarcinoma Cells Displaying Hyperphosphorylated AKT-1 and Synergizes with Conventional Chemotherapy. Oncotarget.

[38] Wilson J., Kunnimalaiyaan S., Gamblin T. (2014). MK2206 inhibits hepatocellular carcinoma cellular proliferation via induction of apoptosis and cell cycle arrest. J. Surg. Res..

[39] Grabinski N., Ewald F., Hofmann B.T., Staufer K., Schumacher U., Nashan B., Jücker M. (2012). Combined targeting of AKT and mTOR synergistically inhibits proliferation of hepatocellular carcinoma cells. Mol. Cancer.

[40] Ewald F., Nörz D., Grottke A., Bach J., Herzberger C., Hofmann B.T., Nashan B., Jücker M. (2015). Vertical Targeting of AKT and mTOR as Well as Dual Targeting of AKT and MEK Signaling Is Synergistic in Hepatocellular Carcinoma. J. Cancer.

[41] Yamashita T., Koshikawa N., Shimakami T., Terashima T., Nakagawa M., Nio K., Horii R., Iida N., Kawaguchi K., Arai K. (2021). Serum Laminin γ2 Monomer as a Diagnostic and Predictive Biomarker for Hepatocellular Carcinoma. Hepatology.

[42] Hashiba T., Yamashita T., Okada H., Nio K., Hayashi T., Asahina Y., Hayashi T., Terashima T., Iida N., Takatori H. (2020). Inactivation of Transcriptional Repressor Capicua Confers Sorafenib Resistance in Human Hepatocellular Carcinoma. Cell. Mol. Gastroenterol. Hepatol..

[43] Hayashi T., Yamashita T., Okada H., Nio K., Hara Y., Nomura Y., Hayashi T., Asahina Y., Yoshida M., Oishi N. (2017). Sporadic PCDH18 somatic mutations in EpCAM-positive hepatocellular carcinoma. Cancer Cell Int..

[44] Yamashita T., Kaneko S. (2021). Liver cancer stem cells: Recent progress in basic and clinical research. Regen. Ther..

[45] Nagano M., Hoshino D., Toshima J., Seiki M., Koshikawa N. (2020). NH 2 -terminal fragment of ZF21 protein suppresses tumor invasion via inhibiting the interaction of ZF21 with FAK. Cancer Sci..

[46] Scrucca L., Fop M., Murphy T.B., Raftery A.E. (2016). mclust 5: Clustering, Classification and Density Estimation Using Gaussian Finite Mixture Models. R J..

