# Supplementary material for: Expression of Cancer Stem Cell Markers EpCAM and CD90 Is Correlated with Anti- and Pro-Oncogenic EphA2 Signaling in Hepatocellular Carcinoma
Source: Int J Mol Sci. 2021 Aug 11;22(16):8652. doi: 10.3390/ijms22168652 (PMC8395527; doi:10.3390/ijms22168652)
Supplement: Supplementary file 1 [file ijms-22-08652-s001.zip › FiguresS3.pdf]

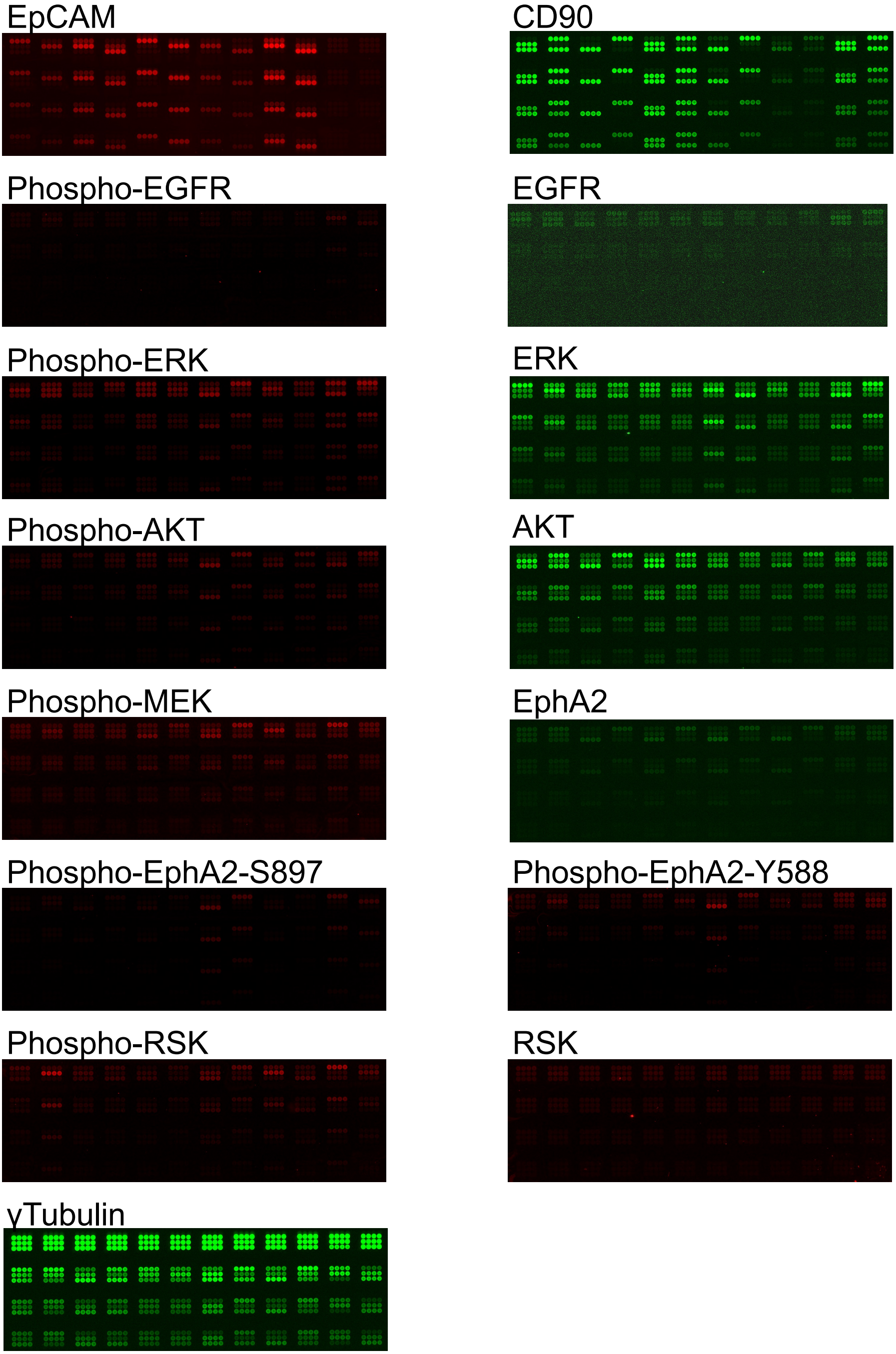
EpCAM
CD90
Phospho-EGFR
EGFR
Phospho-ERK
ERK
Phospho-AKT
AKT
Phospho-MEK
EphA2
Phospho-EphA2-S897
Phospho-EphA2-Y588
Phospho-RSK
RSK
γTubulin
