# Supplementary figures and images for: Expression of Cancer Stem Cell Markers EpCAM and CD90 Is Correlated with Anti- and Pro-Oncogenic EphA2 Signaling in Hepatocellular Carcinoma
Source: Int J Mol Sci. 2021 Aug 11;22(16):8652. doi: 10.3390/ijms22168652 (PMC8395527; doi:10.3390/ijms22168652)

CD90

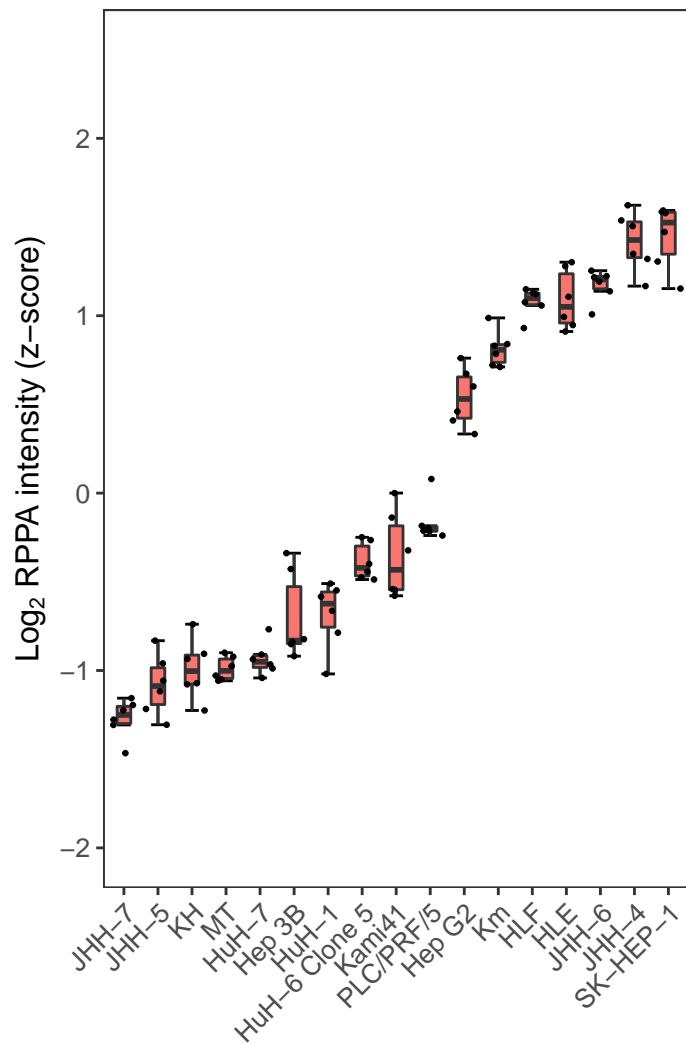

EpCAM

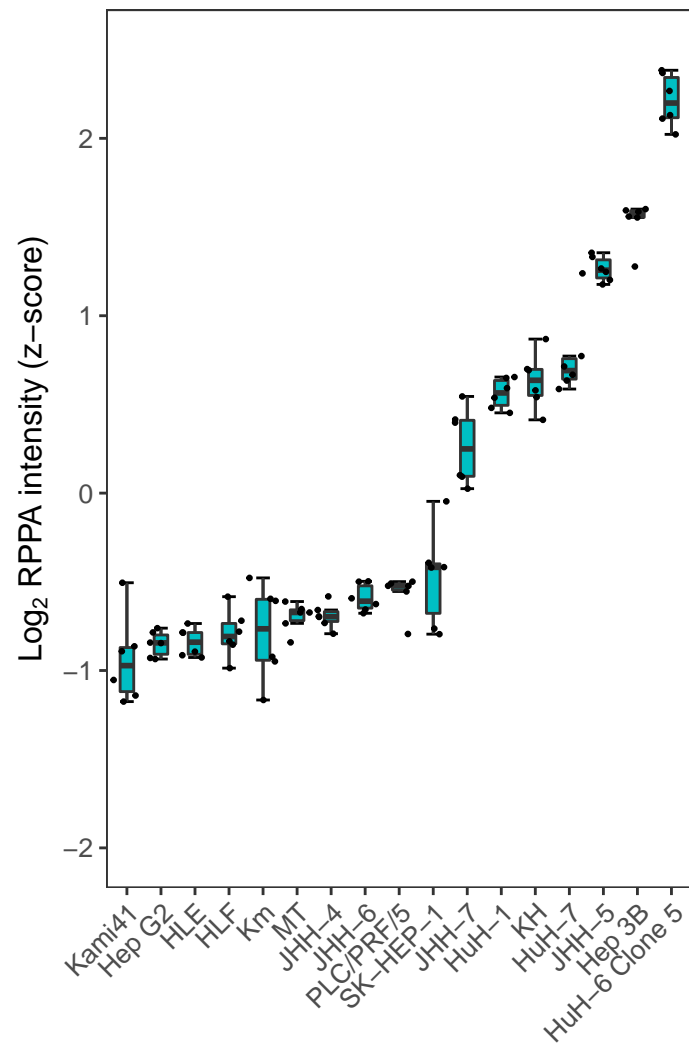

Supplement: Supplementary file 1 [file ijms-22-08652-s001.zip › FigureS1.pdf]

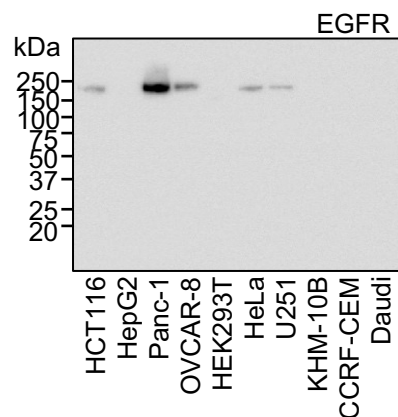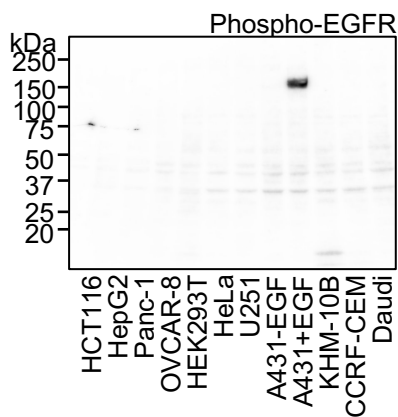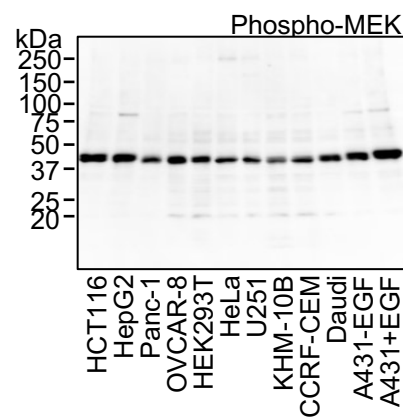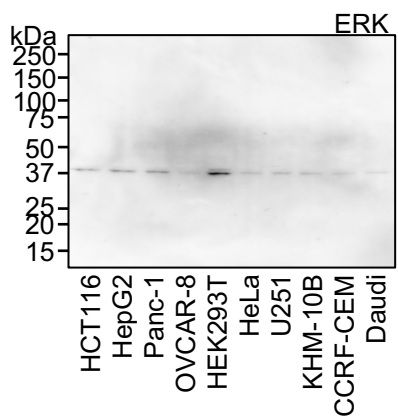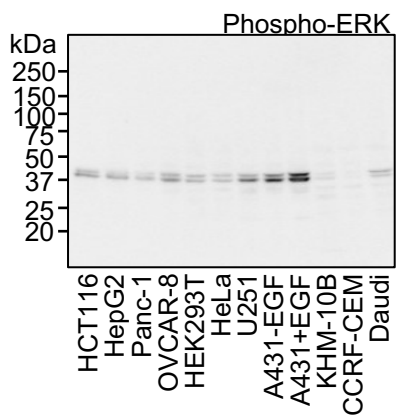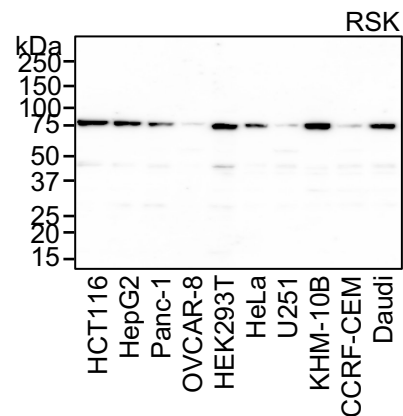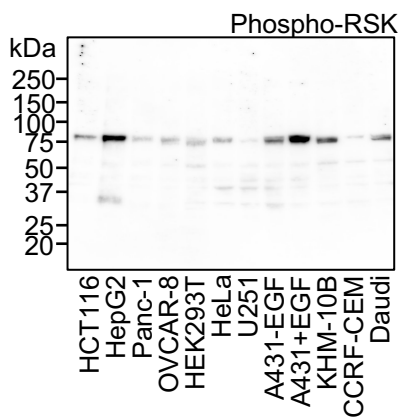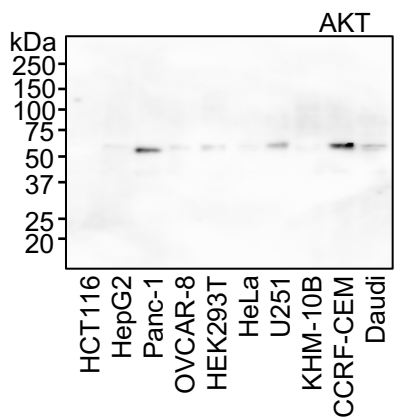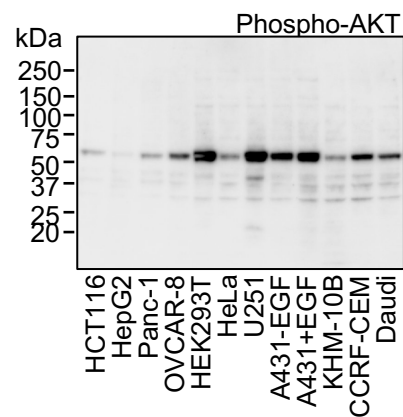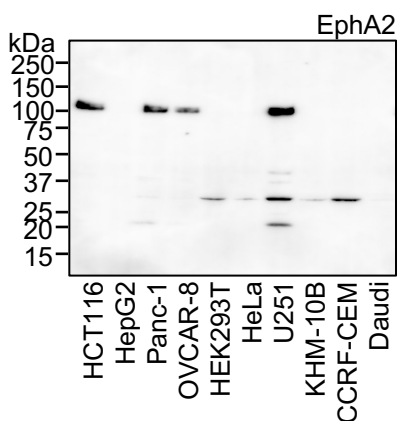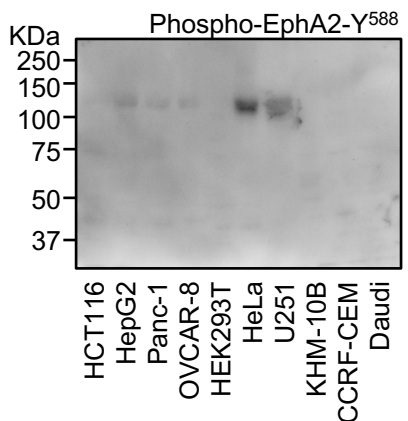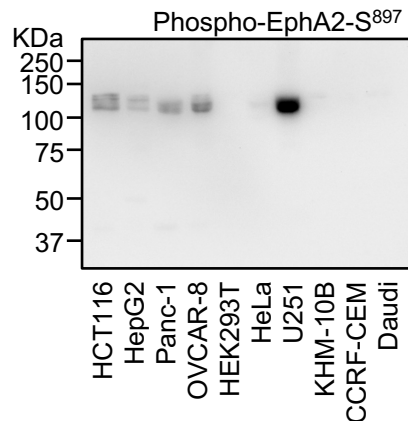

Supplement: Supplementary file 1 [file ijms-22-08652-s001.zip › FiguresS2.pdf]
